# The Combat against National Deterioration

**Published:** 1910-07-30

**Authors:** 


### SPECIAL ARTICLES.

### THE COMBAT AGAINST NATIONAL DETERIORATION.

### II.—LESSONS LEARNT IN THE OUT-PATIENT DEPARTMENT.

The thorough sifting of the applicants for relief in the out-patient department, accomplished by means of the lady almoners, has already laid bare some interesting facts relating to the health of the wage-earning classes. One of the most striking of these is the effect of legislation, based on knowledge. Almost all the improvements which have been accomplished in the supply of pure water and in the disposal of sewage and refuse, improvements which have brought down the death rate in large towns to a figure unprecedented in the annals of vital statistics, are based on the knowledge acquired by dealing with large masses of patients in the voluntary hospitals. The invitation extended by the hospitals to one and all to come and be cured has been responded to with ever-increasing and even embarrassing readiness. Certain facts relating to the spread of disease have thus been thrown into high relief, and for many of the most conspicuous dangers to public health a remedy has been found. But the hospitals have done far more than rescue the population from epidemic disorders due to dirt. They have exercised powerful pressure through the experience gained in the treatment of workmen in favour of fair compensation for accidents. The Employers' Liability Bill was possible mainly because the effects of accident upon the wage-earning class was continually brought under the notice of influential and impartial observers in the wards of the hospitals. The measure has possibly made it harder to find posts for the infirm, but it has removed the heaviest burden on the Samaritan societies, originally founded out of an impulse of sympathy with the hard case of the workman discharged cured but destitute after recovery from an accident.

The Children Act, too, has received a powerful impulse from the testimony forthcoming from the hospitals, and its beneficial results upon child life are gratefully acknowledged by the lady almoner at St. Thomas's. And the more closely the out-patient problem is considered the more clear it becomes that legislation has not so far put forth half its strength in the battle against disease and deterioration.

It would be highly instructive if an attempt could be made to classify the crowds who besiege the out-patient rooms under the causes which have induced their complaints and are hindering their recovery. What factors in their environment have led them to the doctor, and how can these be successfully overcome, not for one individual, but for the long train of sufferers walking in the same direction towards disease and death? Miss Cummins's able report of the people who apply at St. Thomas's throws a good deal of light on the subject. Here is a case of rickets. The child is attending the hospital and being supplied with medicine and nourishment. All is of no avail so long as it lives and sleeps in a damp, dark basement. The family is induced to move, and there is a charitable agency ready to supply the necessary funds to enable them to do so. But why is anyone allowed by law to make money by letting for human habitation a damp, dark cellar? Why do we permit one person to enrich himself at the expense of the community? Think of the money expended to cure that rickety child, who can never really regain health. Think of the labour involved in the hospital, in the almoner's department, in the charitable agency. And all in vain; for the door of the damp, dark cellar has no sooner closed on that rickety child than it opens to receive another family, who in their turn go through the weary round of deteriorated health, resort to the hospital, and rescue by charity. We want a far higher standard among the authorities as to what constitutes a fit habitation. We want powers compulsorily to close these dens which are mere breeding grounds for the out-patient, and no better prelude to vigorous legislation on the subject could be found than the publication by the general hospitals of lists of cases directly due to improper housing.

Again there is the unhealthy employment which need not be unhealthy, except for the greed or carelessness of the employer. We find a girl of eighteen returning four or five times to the hospital with acute rheumatism, recurring within a few months of each "cure." On investigation it appears that her work is that of a packer, and that she stands all day between a hot stove and an open doorway, getting alternately heated and chilled. Or it is a case of pneumonia, and the girl is in a sweetmeat factory, and is compelled night after night to pass in a streaming perspiration straight from her work among the boilers to the icy blasts outside. The almoner may get the patient to change her work, but some other victim steps into her shoes; and is it not transparently true that all this labour and treatment is wasted for want of powers to compel the employer to protect his employees from such grave risks to health? It is not sufficient to cure the patients. It is not sufficient even to put them in the way of retaining their health, though this is finer than cure. The crying need is to stop the inflow. Where the cause of the complaint is a preventable one, as is now admitted in tuberculosis and other complaints, that particular cause needs to be grappled with courageously in the teeth of all the vested interests, and so deservedly great is the influence upon public opinion of the great voluntary hospitals, that few abuses could withstand their united onslaught.

Lastly, the case for a wide extension of colony treatment for many of the chronic sufferers who hang about the out-patient rooms is too strong to be disregarded. For every pound expended out of public money to sequestrate the epileptic, the feeble-minded, and the unrestrained a hundred are saved in the future. The time is ripe for a more thorough handling of these incurable people, always with a view to their own well-being. They are not fit for the strain of competing with normal people. They are happy and busy when the burden of life is eased and suitable occupations and pleasures are found for them. Moreover there is a great awakening to the right methods of dealing with these maimed lives. It is no longer repression or punitive measures which are advocated. It is development of their imperfect faculties along kindly lines which can now be practised for their benefit. Trained workers are ready to undertake the task, and there are facilities for training many more. And every colony of safely tended patients constitutes a barrier set up against the most fatal form of national deterioration—the decadence of race.

These are some of the lessons of the out-patients department, and it behoves us to accept them and act upon them, for they have been costly not in money alone.

